# Antibiotic-resistant *Acinetobacter calcoaceticus-baumannii* complex in the environment: a narrative review

**DOI:** 10.1093/femsec/fiag106

**Published:** 2026-09-16

**Authors:** Yu T Zheng, Danielle Schneiderman, Ayush Kumar, Christine Liu, Santiago Castillo-Ramírez, Izhar U H Khan

**Affiliations:** Agriculture and Agri-Food Canada, Ottawa Research and Development Centre (ORDC), Ottawa, ON K1A 0C6, Canada; University of Western Ontario, London, ON N6A 3K7, Canada; Agriculture and Agri-Food Canada, Ottawa Research and Development Centre (ORDC), Ottawa, ON K1A 0C6, Canada; University of Manitoba. Winnipeg, MB R3T 2N2, Canada; Agriculture and Agri-Food Canada, Lacombe Research and Development Centre, Lacombe, AB T4L 1V8, Canada; National Autonomous University of Mexico, 62210, Mexico; Agriculture and Agri-Food Canada, Ottawa Research and Development Centre (ORDC), Ottawa, ON K1A 0C6, Canada

**Keywords:** *Acinetobacter calcoaceticus-baumannii* complex, environment, antibiotic resistance, review

## Abstract

*Acinetobacter calcoaceticus*–*baumannii* (ACB) complex comprises of both environmental and pathogenic bacterial species. Pathogenic species are responsible for nosocomial infections, which are increasingly difficult to treat due to growing resistance to antibiotics. In addition to nosocomial settings, ACB complex has also been found in other environmental niches, including animals, food, soil, and water, where human activity has contributed to the development of antibiotic resistance. For instance, improper disposal of solid waste and release of wastewater effluent carrying antibiotic-resistant pathogens have introduced antibiotic resistance genes into the natural environment. Additionally, soil and water environments near agricultural operations are hotspots for ACB complex strains and antibiotic resistance. Antibiotic resistant ACB complex are also transmitted through food production system. While antibiotic resistance level in strains from healthy animals is low, it can be highly prevalent on meat. Meat and produce fertilized by manure from antibiotic-treated animals are a pathway for transmission of ACB complex back to human and clinical environments, reflecting the interconnectedness of humans, animals, and the environment. This review has revealed research gaps, particularly on non-*Acinetobacter baumannii* ACB complex species in the environment. Understanding the “One Health” dimension of these important nosocomial pathogens is critical for informing policies on antibiotic use, agriculture, and waste management to reduce and prevent the emergence of antibiotic resistant strains.

## Teaser

ACB Complex and Environment: Review.

## Introduction

The *Acinetobacter* genus is made up of aerophilic Gram-negative bacteria first recovered from soil samples enriched with calcium–acetate minimal medium and named *Micrococcus calcoaceticus* in 1911 (Beijerinck 1911). Since then, numerous species have been recognized within the genus *Acinetobacter*, which comprises a diverse group of environmentally widespread organisms associated with both clinical infections and ecological niches (Dahal et al. 2023, Yehya et al. 2025). In the early days, phenotypic tests used to identify *Acinetobacter* spp. were ineffective in accurately discerning between a closely related group of species that included *A. baumannii, A. calcoaceticus, A. pittii*, and *A. nosocomialis*, thus, the *Acinetobacter calcoaceticus*–*baumannii* (ACB) complex group was proposed to classify these species (Gerner-Smidt et al. 1991). Importantly, phylogenomic analyses have uncovered several cases of misclassification when considering whole genome sequences (WGS), rather than phenotypic tests (Gerner-Smidt et al. 1991, Mateo-Estrada et al. 2019). Since then, other newly discovered species have been added to the group, including *A. seifertii* (Nemec et al. 2015), *A. lactucae*, later found to be synonymous with *A. dijkshoorniae* (Rooney et al. 2016, Dunlap and Rooney 2018), and most recently *A. geminorum* (Wolf et al. 2021). While *A. calcoaceticus* is considered a largely non–pathogenic environmental species and is only rarely associated with human infection, the other members of the ACB complex are often implicated in clinical cases (Cosgaya et al. 2016, Kishii et al. 2016, Mancilla-Rojano et al. 2020). Species from this group are known as a leading cause of human-associated nosocomial (healthcare–associated) infections, including ventilator–associated pneumonia, bloodstream infections, urinary tract infections, meningitis, and wound infections (Yehya et al. 2025). Some members of the ACB complex are also extremely persistent in the hospital and natural environment due to their high tolerance to oxidative stress, desiccation, and disinfection, as well as their ability to form biofilms (Harding et al. 2017).

Currently, various drug therapies are available to treat infections caused by the ACB complex, and are selected based on the severity of infection, the antibiotic resistance of the strain causing it, and the site of infection. First-line treatments include carbapenems and other beta-lactam antibiotics such as broad-spectrum cephalosporins, piperacillin-tazobactam, and ampicillin-sulbactam (Rangel and De-Simone 2024). Aminoglycosides (i.e. gentamicin, tobramycin, amikacin) and trimethoprim-sulfamethoxazole (also known as co-trimoxazole) are used for urinary tract infections (Falagas et al. 2015, Thy et al. 2023). If a strain is resistant to first-line treatments, then second-line treatments such as polymyxins and tetracyclines are employed (Rangel and De-Simone 2024). Concerningly, resistance to these antimicrobial agents is growing in *Acinetobacter*. Strains are classified as multidrug resistant (MDR) if they have acquired non-susceptibility to one or more agents in three or more antimicrobial categories, and extensively drug resistant (XDR) if they are non-susceptible to one or more agents in all but two or fewer categories (Magiorakos et al. 2012, Xie et al. 2018). MDR, XDR, and carbapenem-resistant strains are of great concern to the medical community due to the challenges associated with treating those infections. The World Health Organization has designated carbapenem-resistant *A. baumannii* as a critical priority target for research and development of new antimicrobial agents (Sati et al. 2025). Furthermore, some ACB complex isolates recovered from environmental sources have been found to be genetically related to clinical clones, raising concerns about the potential exchange of pathogens between environmental and clinical settings (Seruga Music et al. 2017, Wilharm et al. 2017, Furlan et al. 2018, Tavakol et al. 2018). Documented cases of community-acquired infections also indicate that there are reservoirs of antibiotic-resistant pathogenic species in the environment, which pose a growing threat to public health (Eveillard et al. 2013, Souhail et al. 2024).

**Figure 1:**
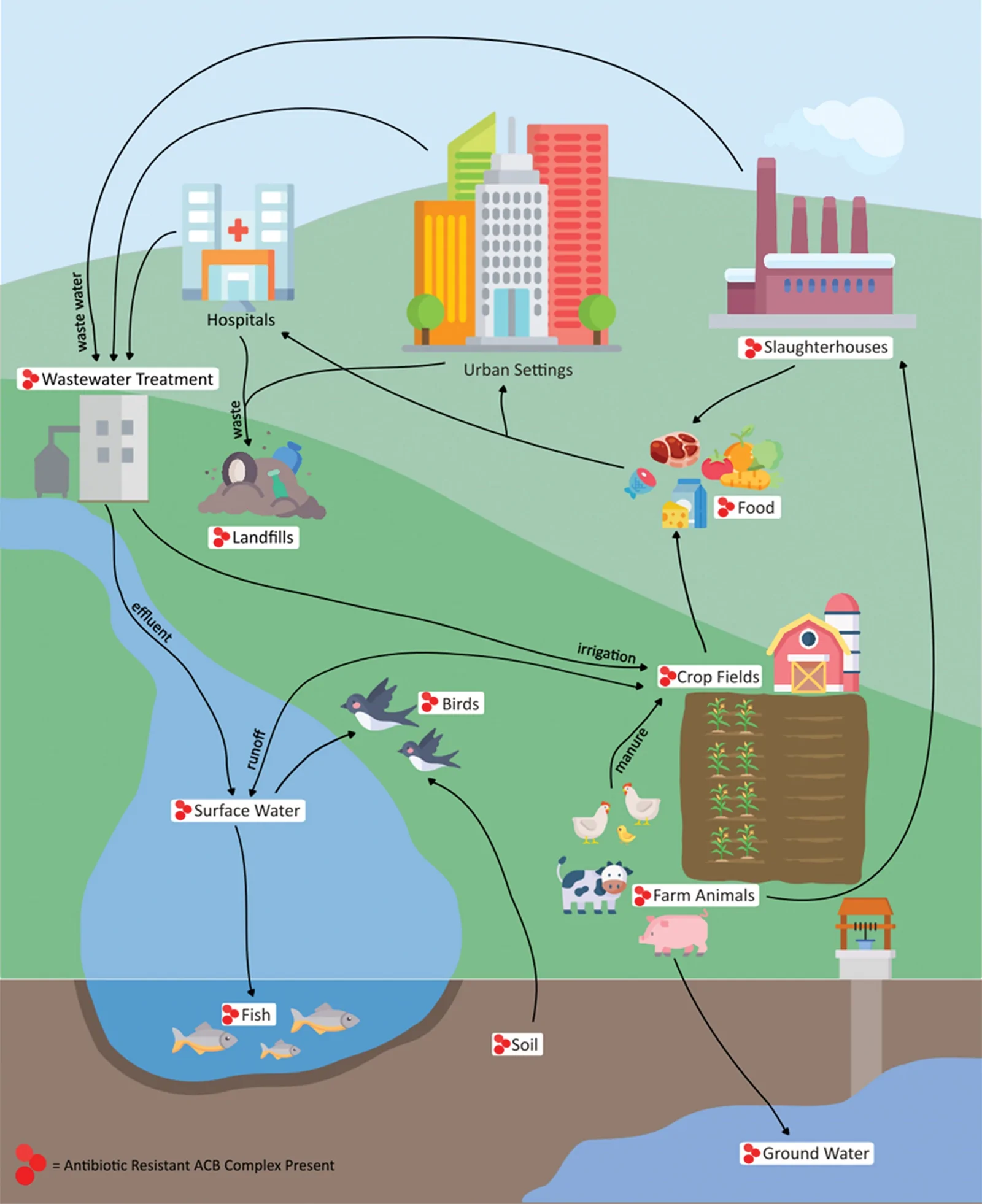
Diagram summarizing the distribution and potential transmission pathways of antibiotic ACB complex in non-clinical environments. (Created using Inkscape with images from Flaticon.com).

The purpose of this article is to review the distribution of ACB complex species in environmental sources, including soil, water, animals, and food, with a focus on antibiotic-resistant environmental isolates. This topic will be explored through a “One Health” perspective, which recognizes the close relationship among human, animal, and environmental health and food safety. Specifically, human activities like the widespread use of antibiotics in clinical and agricultural settings may contribute to the development of antibiotic resistance in ACB complex strains in the environment and the expansion of antibiotic resistance genes (ARGs) (Kraemer et al. 2019). Moreover, environmental compartments such as wastewater and agricultural soil can act as interfaces for horizontal gene transfer and the movement of resistant strains among environmental, animal, and human populations (Sardar et al. 2023, Li et al. 2026) (Fig. 1). Understanding the prevalence, diversity, and transmission pathways of antibiotic-resistant ACB complex outside of clinical settings is critical for assessing their potential to acquire and disseminate resistance and the risks they pose to public health.

## Aquatic environment

ACB complex species have been isolated from diverse aquatic systems, including ponds, rivers, lakes, and drinking water, around the world, (Girlich et al. 2010, Narciso-Da-Rocha et al. 2012, Tacão et al. 2012, Pindi et al. 2013, Goswami et al. 2015, Benoit et al. 2024, Tobin et al. 2024) (Table 1.1). Some species are widespread in the natural aquatic environment, such as *A. calcoaceticus*, which was one of the most frequently identified bacterial species in a non-polluted river in Portugal (Tacão et al. 2012). Meanwhile, the distribution of other ACB complex species has been less well elucidated (Benoit et al. 2024). Although a study in Lebanon recovered several *A. baumannii* and *A. pittii* from a diverse range of water sources, it is not clear whether these isolates constitute a natural presence or are a result of human contamination (Rafei et al. 2015).

**Table 1:** ACB complex species isolated from various environments listed based on country of origin, sampling source, detection methodology, and antimicrobial resistance.

	Species	Country / region	Source / sample type	Detection method	Antimicrobial resistance
**1. Aquatic environments**					
Natural water	*A. calcoaceticus*	Portugal	Non-polluted river	Culture-based	Not reported
Water	*A. baumannii, A. pittii*	Lebanon	Artesian well, other sources unspecified	Culture-based	Not reported
Agricultural water	*A. baumannii, A. pittii, A. nosocomialis*	Canada	Rivers, streams, drinking water near agricultural land	Culture-based	Not specified; prevalent in fall during manure application
Agricultural water	*A. baumannii, A. lactucae*	Côte d’Ivoire	Manmade lake near vegetable fields	Culture-based	Not reported
Agricultural water (groundwater)	*A. baumannii, A. pittii/oleivorans, A. seifertii*	China	Groundwater, swine farming village	Culture-based	MDR: tetracyclines, polymyxins, fluoroquinolones
Agricultural water (surface)	*A. baumannii*	China	River adjacent to livestock farms	Culture-based	Mostly susceptible; one isolate resistant to tetracycline
Urban river	*A. baumannii, A. nosocomialis*	South Africa	Rivers near urban and rural activities	Culture-based	MDR (48.7% *A. baumannii*)
Urban river	*A. baumannii*	France	Seine River	Antibiotic-supplemented media	MDR; carbapenem-resistant
Urban river	*A. baumannii*	Brazil, Ecuador	Urban rivers	Antibiotic-supplemented media	MDR; carbapenem-resistant
Urban river	*A. calcoaceticus*	Croatia	Polluted urban river	ESBL selective media	ESBL-producing; β-lactam/ β-lactamase inhibitors and polymyxin B resistant
Urban river	*A. baumannii*	Austria/Europe	Danube River	Culture-based	MDR; high cephalosporin resistance
Urban surface water	*A. baumannii*	Bangladesh	Surface water	Culture-based	MDR (majority); ∼25% XDR
Municipal dam water	*A. baumannii*	South Africa	Municipal dam	Carbapenemase selective media	MDR in >50% of isolates
**2. Wastewater treatment plants (WTPs)**					
Municipal WTP	*A. baumannii*	Croatia	WTP downstream; effluent and surface water	Culture-based; MDR selective media; sequence typing	MDR; XDR; same ST as clinical isolates
Municipal WTP	*A. pittii, A. nosocomialis, A. baumannii*	Canada	All stages of chlorination treatment	Culture-based	Survived disinfection; resistance not fully specified
Municipal WTP	*A. baumannii*	Romania	WTP effluent and adjacent rivers	Culture-based; ESBL selective and Carbapenemase selective media	MDR; variable profiles across sites and timeframes
Municipal WTP	*A. baumannii*	Poland	Treated municipal wastewater	Culture-based; MDR selective media; molecular (carbapenem resistance genes)	Carbapenem-resistant
Slaughterhouse WTP	ACB complex	Germany	Slaughterhouse wastewater treatment	Culture-based	Different ARG profiles vs. municipal WTP isolates
**3. Soil environments**					
Natural/Pristine Soil	*A. baumannii*	Not specified	Pristine soil (decomposing material)	Long-term ecological study; genomic	bla_OXA-51_-like genes
Natural soil	*A. calcoaceticus, A. baumannii, A. pittii*	Lebanon	Urban and agricultural soil	Culture-based	Not specified
Natural soil	*A. baumannii*	Ireland	Grass	Culture-based; genomic	Non-resistant; novel clones
Urban soil	*A. baumannii, A. pittii*	Hong Kong	Urban soil	Genomic	Not specified
Urban soil	*A. baumannii*	Morocco	Soil near hospital	Culture-based	Resistant to cephalosporins, β-lactam/β-lactamase inhibitor combination agents, aminoglycosides
Urban soil	*A. baumannii*	Iraq	City soil	Culture-based	Resistant to cephalosporins, aminoglycosides, fluoroquinolones
Urban soil	*A. baumannii*	Jordan	Park and city soil	Culture-based	Antibiotic-resistant; one MDR strain from park soil
Urban soil	*A. baumannii*	India	City soil	Culture-based	MDR: carbapenems, fluoroquinolones, tetracyclines, aminoglycosides
Urban soil (landfill)	*A. baumannii*	Croatia	Illegal landfills and dump sites	Culture-based; sequence typing	MDR; related to clinical strains
Urban soil	*A. calcoaceticus*	South Africa	Town soil	Culture-based	Resistant to cephalosporin, carbapenem, tetracycline
Agricultural soil	*A. pittii, A. baumannii*	Korea	Agricultural fields (streptomycin used as pesticide)	Culture-based	Resistant to streptomycin, colistin
Agricultural soil	*A. baumannii*	Not specified	Pig slurry-treated soil	Experimental study (2 years post-treatment)	Sulfonamide-resistant
Agricultural soil	*A. seifertii*	Brazil	Corn field soil	Culture-based	MDR: aminoglycosides, cephalosporins, tetracyclines
Agricultural soil	*A. baumannii*	Brazil	Agricultural soil	Culture-based	MDR: extended-spectrum cephalosporins, carbapenems, tetracyclines
**4. Animals**					
Companion animals	*A. baumannii*	Reunion Island	Healthy companion animals	Culture-based; clonal lineage typing	Susceptible to most antibiotics
Companion Animals	*A. pittii, A. calcoaceticus*	Not specified	Healthy canine skin	Culture-based	Not specified
Wild birds	*A. baumannii, A. pittii*	Poland	White stork nestlings	Culture-based	Susceptible
Wild birds	*A. baumannii, A. pittii*	Europe	Wild birds	Culture-based	Limited resistance
Captive/wild migratory birds	*A. baumannii, A. seifertii*	Brazil	Captive and wild aquatic birds (zoo)	Culture-based; sequence typing	MDR; carbapenem-resistant
Urban birds	*A. baumannii, A. pittii, A. nosocomialis*	Not specified	Wild pigeon droppings	Culture-based; molecular (carbapenemase genes)	Carbapenemase-encoding genes detected
Fish	*A. baumannii, A. pittii, A. calcoaceticus*	South Africa	Fish from municipal dam	Culture-based	MDR *A. baumannii*; others not tested
Fish (aquaculture)	*A. pittii*	India	Carp farm outbreak	Culture-based	Resistant to ceftazidime, cefotaxime
Farm animals	*A. baumannii*	Germany	Chicken and geese choana swabs; duck hatchery air	Culture-based	Resistance to aminoglycosides only in two isolates
Farm animals	*A. baumannii*	Korea	Pigs and cows	Culture-based	One isolate resistant to tobramycin
Farm animals	*A. baumannii*	Germany	Cow nasal, rectal, fecal samples	Culture-based	Susceptible to clinical antibiotics; resistant to ceftiofur (veterinary)
Farm animals	*A. baumannii*	Scotland	Pig and cow fecal samples	Culture-based	Susceptible
Poultry	ACB complex	China	Fecal and environmental samples, poultry farms	Culture-based	8.9% resistant to tigecycline and tetracycline
Poultry manure	*A. baumannii*	Not specified	Meconium (one-day-old chicks)	Culture-based	Resistant to ciprofloxacin, fluoroquinolones, tetracyclines, trimethoprim-sulfamethoxazole
Pig/cow manure	*A. baumannii*	Not specified	Pig and cow feces	Culture-based; molecular	Rare carbapenem resistance (despite no carbapenem use in livestock)
**5. Food: meat and slaughterhouses**					
Meat production	*A. baumannii, A. pittii, A. calcoaceticus*	Germany	Chicken and pig meat production	Culture-based	High cephalosporin resistance; occasional fluoroquinolone, colistin, carbapenem resistance; ST345 with mcr-4.3
Slaughterhouse	*A. baumannii*	Korea	Cattle and pig carcasses	Culture-based; sequence typing	Resistant to ampicillin-sulbactam, tetracycline or tobramycin
Slaughterhouse	*A. baumannii, A. pittii, A. calcoaceticus*	South Africa	Slaughterhouse equipment and surroundings	Culture-based; sequence typing (ST1, ST2)	MDR (majority); overlapping with hospital clones
Retail meat	*A. nosocomialis, A. seifertii, A. pittii, A. baumannii*	Korea	Local and imported meats	*Campylobacter* selective conditions	Two MDR A. nosocomialis; others resistant to 1–2 antibiotics
Retail meat	*A. baumannii, A. pittii, A. seifertii, A. calcoaceticus, A. nosocomialis*	Portugal	Various meat types	Culture-based	38.7% MDR; one XDR *A. pittii*
Retail meat/fish	*A. baumannii, A. pittii, A. dijkshoorniae*	Peru, Lebanon, India	Meat and fish	Culture-based	Susceptible to clinically relevant antibiotics
Retail meat	*A. baumannii*	Saudi Arabia	Camel, chicken, sheep, cow meat	Culture-based	Resistant to tetracycline, gentamicin, tobramycin; low cephalosporin/carbapenem resistance
Retail meat	*A. baumannii*	Iran	Meat samples	Culture-based	High resistance to gentamicin, tetracycline, ciprofloxacin, trimethoprim-sulfamethoxazole; low carbapenem resistance
Seafood	*A. baumannii*	Iran	Fish, lobster, shrimp	Culture-based	Resistant to gentamicin, tetracycline, trimethoprim-sulfamethoxazole
Retail meat	*A. baumannii*	France, Switzerland	Various meats	Culture-based	Mostly susceptible; sporadic ciprofloxacin, colistin, tetracycline resistance
**6. Food: dairy products**					
Raw milk	*A. baumannii, A. calcoaceticus*	Israel	Raw milk from farms	Culture-based	Not specified; strong lipolytic activity
Raw milk, cheese, cream	*A. baumannii, A. calcoaceticus*	Egypt	Raw milk (not in pasteurized milk), cheese, cream	Culture-based	Not specified
Raw milk	*A. baumannii*	Egypt	Retail raw milk	Culture-based; molecular (*bla*_OXA-23_, *bla*IMP, *bla*NDM)	Resistant to cefotaxime, ampicillin-sulbactam, levofloxacin; carbapenem resistance
Raw milk	*A. baumannii*	Korea	Raw milk	Culture-based	Mostly susceptible; some resistant to tetracyclines, trimethoprim-sulfamethoxazole, ampicillin-sulbactam, cephalosporins, aminoglycosides
**7. Food: fruits and vegetables**					
Agricultural crops	*A. seifertii, A. pittii*	Korea	Crops from agricultural environments	Culture-based; molecular (*emrA*/*emrB* efflux genes)	Resistant to colistin; *emrA*/*emrB* positive
Fresh produce	*A. calcoaceticus, A. baumannii*	France	Unwashed fruits and vegetables	Culture-based	Resistant to cefotaxime, ceftazidime; rare imipenem resistance in *A. calcoaceticus*
Fresh produce	*A. pittii, A. calcoaceticus*	Algeria	Fruits and vegetables	3rd-gen cephalosporin selective media	Cephalosporin-resistant; otherwise wild-type profile
Organic produce	*A. baumannii, A. calcoaceticus*	Spain	Organic produce	Cefotaxime and carbapenem selective media	MDR (4 strains); resistant to multiple antibiotics
Ready-to-eat salads	*A. pittii, A. calcoaceticus, A. geminorum, A. lactucae*	Portugal	Ready-to-eat salads	Cefotaxime selective media; ESBL gene sequencing	ESBL genes: *bla*ACC, *bla*SHV, *bla*DHA, *bla*VEB
Unwashed fruit/lettuce	*A. calcoaceticus, A. pittii, A. baumannii, A. seifertii, A. nosocomialis*	Portugal	Unwashed fruit and lettuce	Culture-based	MDR and XDR isolates; A. calcoaceticus identified as endophyte
Retail produce	*A. baumannii, A. pittii*	Jordan	Fresh produce from retail markets (fruits, radish)	Culture-based; sequence typing (ST2)	Mostly susceptible; 4 XDR isolates; two novel STs; one ST2 (global clinical clone)

### Agricultural water environments

A Canadian study sporadically isolated *A. baumannii, A. pittii*, and *A. nosocomialis* isolates in rivers, streams, and a drinking water intake source near agricultural land (Benoit et al. 2024). ACB complex species were most prevalent in the fall, when manure application occurs, and heavy rainfall facilitates runoff into nearby rivers. Use of animal manure to fertilize crop fields may have introduced ACB complex species into bodies of water near crop farming. Similarly, *A. baumannii* and *A. lactucae* were isolated from a manmade lake near vegetable fields in Côte d’Ivoire, where farmers used chicken manure as fertilizer (Yebouet et al. 2025). Although these studies were conducted in different agricultural (row-crop farmland vs. vegetable-farming) settings, the shared use of manure as fertilizer suggests that manure application may represent a common pathway for introducing ACB complex species into nearby water bodies, independent of the specific farming or geographical context. Additionally, aquatic environments near livestock farming operations have been studied as potential reservoirs of antibiotic-resistant ACB complex due to antibiotic overuse in animal husbandry (Tsai et al. 2018, Benoit et al. 2024). These antibiotic compounds and clinically relevant microbes are able to spread from agricultural settings into the surrounding water sources (Khmaissa et al. 2024). For instance, in the groundwater of a swine farming village in China, *Acinetobacter* spp. were highly prevalent, as compared to non-swine-farming villages (Gao et al. 2023). The swine farm isolates included *A. baumannii, A. pittii*/ *A. oleivorans*, and *A. seifertii*, which were MDR to tetracyclines, polymyxins, and fluoroquinolones. A similar effect can be seen in surface water, where parts of a river in China adjacent to livestock farming operations were hotspots for *A. baumannii* isolation compared to other parts of the river (Tsai et al. 2018). However, compared to the study on groundwater, these isolates were mostly susceptible to antibiotics, with only one isolate resistant to tetracycline. This difference may be explained by the fact that environmental contaminants are more concentrated in groundwater and can remain there for a long time before being discharged, allowing for the development of antibiotic-resistant microbes (Irvine et al. 2024). These two studies demonstrate that livestock farming can yield different resistance patterns depending on the water type (groundwater vs. surface water) where confined water concentrate and retain resistant strains, whereas flowing water dilutes and disperses contaminants, limiting sustained selective pressure.

## Urban water environments

ACB complex species have been found in water bodies near human activity and especially in urbanized areas. A study from South Africa that sampled water from rivers exposed to both urban and rural anthropogenic activities identified 48.7% of *Acinetobacter* isolates as *A. baumannii* and 2.7% as *A. nosocomialis* (Adewoyin et al. 2024). In particular, the current research indicates that ACB complex species found in urban surface water can display high levels of antibiotic resistance despite their environmental origin. For instance, MDR and carbapenem resistant *A. baumannii* have been isolated from the Seine River in downtown Paris, as well as urban rivers in Brazil and Ecuador using antibiotic-supplemented media (Girlich et al. 2010, Turano et al. 2016, Sotomayor et al. 2024). Similarly, extended-spectrum β-lactamase (ESBL) producing *Acinetobacter* spp., including *A. calcoaceticus* resistant to β-lactam/β-lactamase inhibitor combination agents and polymyxin B, were isolated from a heavily polluted urban river in Croatia with ESBL selective media (Maravić et al. 2015). Finally, a South African study using carbapenem resistance selective media found multidrug resistance in over half of *A. baumannii* isolates from municipal dam water (Anane et al. 2019). Due to the use of antibiotic resistance selective media in these studies, it’s difficult to discern the prevalence of highly resistant strains. Studies which don’t rely on the use of antibiotic supplemented media have also isolated antibiotic-resistant strains in urban surface water. In the Danube River, a high prevalence of cephalosporin resistance was found in the *Acinetobacter* isolates, and an MDR *A. baumannii* strain was also recovered (Kittinger et al. 2017). In another study, 32% of surface water samples in Dhaka, Bangladesh, tested positive for *A. baumannii* (Hossain et al. 2025). Concerningly, the majority of isolates were MDR and almost a quarter were XDR. The consistent detection of MDR/XDR isolates across geographically diverse sites, despite variation in isolation methodology using antibiotic supplemented vs. non-selective media, suggests that urbanization constitutes a common underlying driver. This association is likely related to sewage discharge, surface runoff, and the elevated human population density characteristic of urban settings, which collectively increase the concentration of both bacterial loads and antibiotic residues in receiving waters. Overall, the discovery of ACB complex species across aquatic environments, particularly in settings influenced by agriculture, wastewater discharge, and urbanization, clearly shows the impact of human activity on the dissemination of antibiotic resistant ACB complex species in aquatic ecosystems.

### Wastewater treatment plants

Wastewater treatment plants (WTPs) process wastewater from urban and industrial settings to remove pathogens and other pollutants before discharging the effluent back into the environment. However, ACB complex species, including *A. pittii, A. nosocomialis*, and *A. baumannii*, were shown to survive in wastewater and were found in all stages of the disinfection by chlorination treatment process in a Canadian study (Benoit et al. 2024). In-house WTPs of slaughterhouses in Germany were similarly ineffective in removing the ACB complex species from treated wastewater (Savin et al. 2024) (Table 1.2). Additionally, *A. baumannii* isolates collected from downstream of a WTP in Zagreb, Croatia, were classified as the same sequence type as those concurrently collected from clinical cases in a nearby hospital, indicating genetic relatedness between clinical and environmental populations. However, this finding alone does not establish the source or direction of transmission between these reservoirs (Seruga Music et al. 2017). Although *A. baumannii* was found to be 97.5–99.7% eliminated during wastewater treatment, water collected downstream of the WTP contained higher levels of *A. baumannii* than upstream (Hubeny et al. 2022). Furthermore, a study also found *A. baumannii* isolates were most prevalent in effluent compared to earlier stages of treatment, suggesting that WTPs may promote *A. baumannii* selection (Benoit et al. 2024). Moreover, *A. baumannii* isolates have been found to be able to multiply and persist in effluent wastewater for a period of over 50 days, indicating a high survival rate and potential to infiltrate into surrounding natural environment (Hrenovic et al. 2016).

In Romania, MDR *A. baumannii* was isolated from WTP effluent and the rivers where the effluent was discharged (Gheorghe-Barbu et al. 2024). Additionally, the antimicrobial resistance profiles of isolates from different sampling sites and timeframes showed considerable differences, possibly reflecting the influent received by the WTP (Gheorghe-Barbu et al. 2024). This is seen in other studies as well, for instance, ACB complex species from slaughterhouse WTPs carried different ARGs and prevalence of phenotypic resistance compared to municipal WTPs (Savin et al. 2024). In water associated with municipal WTPs, ACB complex species resistant to clinically important antibiotics can be readily isolated using selective media. In Croatia, MDR and XDR *A. baumannii* was found in effluent wastewater and downstream surface water using MDR selective media (Hrenovic et al. 2016, Seruga Music et al. 2017). Similarly, a Polish study which also used MDR selective media found *A. baumannii* isolates carrying carbapenem resistance genes in treated municipal wastewater samples (Hubeny et al. 2022). Although these studies help elucidate the ARG profiles of ACB complex strains found in WTP associated water, the actual prevalence of antibiotic-resistant strains is still unclear due to the use of MDR selective media, which overinflates the rate of resistance in recovered isolates. However, there is research suggesting WTP processing could cause a selective increase in the antibiotic resistance in *Acinetobacter* spp., as prevalence of resistance in isolates from effluent wastewater was higher than isolates in influent wastewater, despite an overall decrease in the total bacterial population (Zhang et al. 2009). Additionally, wastewater is a prime location for ARG transfer due to the co-occurrence of ARG carrying hosts, allowing pathogens such as *A. baumannii* to acquire and spread new ARGs in the environment (Lund et al. 2025). Although there is geographic diversity among these studies, a consistent pattern has been observed where WTPs reduced overall bacterial abundance but complete elimination of the ACB complex species was not achieved. Moreover, the resistant isolates were frequently enriched in effluent relative to influent. This suggests that treatment processes such as chlorination may exert a selective effect, preferentially inactivating susceptible strains while permitting resistant subpopulations to persist, thereby concentrating rather than eliminating resistance determinants within the treated effluent. The clonal overlap observed between clinical and downstream environmental isolates in Croatian study suggests an epidemiological association between clinical and environmental reservoirs. However, shared clonal types alone cannot determine the direction of movement or transmission. Therefore, WTPs can be considered a potential point of interface between these reservoirs.

## Soil environment

Multiple species in the ACB complex group have been shown to colonize and play ecological roles in soil environments (Dahal et al. 2023) (Table 1.3). For instance, *A. calcoaceticus, A. baumannii*, and *A. pittii* isolated from crude oil and diesel contaminated soil were shown to have bioremediation potential in several studies (Méndez et al. 2017, Ho et al. 2020, Zhang et al. 2021, Kumar et al. 2023, Dohare et al. 2024). *A. calcoaceticus* isolates from the rhizosphere of plants have also displayed plant growth-promoting properties (Kour et al. 2023). Novel clones of non-resistant *A. baumannii* have also been described in grass in Ireland (Mateo-Estrada et al. 2023). In human-associated soil environments, Rafei et al. (2015) found *A. pittii, A. baumannii*, and *A. calcoaceticus* in soil samples from various urban and agricultural areas in Lebanon. A recently published long-term ecological study found a natural population of *A. baumannii* living in pristine soil environments rich in decomposing material (Wilharm et al. 2026). A diverse collection of *bla*_OXA-51_-like β-lactamase encoding genes were found in the natural population, suggesting that a baseline reservoir of resistance-associated genes exists in ACB complex populations independent of anthropogenic antibiotic pressure, showing that not all resistance determinants detected in soil isolates can be attributed to human activity. This distinction is important for interpreting the urban and agricultural soil findings, where resistance is more directly linked to human antibiotic use.

### Urban soil

ACB complex strains are readily isolated from urban soil environments. In one of the first widespread studies using genomic identification methods, *A. baumannii* and *A. pittii* were isolated from soil samples collected around Hong Kong (Houang et al. 2001). Interestingly, another study also found *A. baumannii* and *A. pittii*, but did not isolate any *A. nosocomialis* from urban soil, despite finding *A. nosocomialis* on nearby urban surfaces such as shopping carts and public bathroom appliances (Ababneh et al. 2022a). This suggests that unlike *A. baumannii* and *A. pittii*, soil may not be a habitat of *A. nosocomialis*.

Urban soil can also harbour antibiotic-resistant ACB complex strains. For instance, *A. baumannii* isolated from soil near a hospital in Morocco showed resistance to cephalosporins, β-lactam/β-lactamase inhibitor combination agents, and aminoglycosides (El Hafa et al. 2019). However, the prevalence of resistance was significantly less than that of clinical isolates from the hospital, and no carbapenem resistance was found (El Hafa et al. 2019). Similar findings were reported from a research group in Iraq, where *A. baumannii* isolates from city soil had resistance to cephalosporins, aminoglycosides, and fluoroquinolones, but once again the prevalence of antibiotic resistance was much lower than in clinical isolates (Hassan et al. 2020). In both studies, MDR strains were exclusively isolated from clinical environments. Rare instances of MDR *A. baumannii* have been isolated from urban soil in other studies. In Jordan, one novel strain isolated from park soil was classified as MDR, while all other isolates from park and city soil were not highly resistant (Ababneh et al. 2022a). Similarly in India, two *A. baumannii* strains isolated from city soil were MDR to carbapenems, fluoroquinolones, and tetracyclines and aminoglycosides (Suresh et al. 2022). While the Moroccan and Iraqi studies did not find high levels of resistance in *A. baumannii* from urban soil in comparison to clinical isolates, studies from Jordan and India did report MDR strains isolated directly from city and park soil. This suggests that proximity to discrete contamination sources, rather than urban soil as a homogeneous exposure category, may be a more accurate predictor of MDR strain distribution. Interestingly, MDR *A. baumannii* isolates related to clinical strains were recovered near illegal landfills and dump sites in Croatia (Hrenovic et al. 2014, 2017). These findings suggest improperly disposed clinical waste may be a way antibiotic-resistant isolates or antibiotic resistance genes which soil-dwelling acinetobacters acquire, can enter the urban soil environment. Currently, studies screening for antibiotic resistance in ACB complex species other than *A. baumannii* in soil are lacking. One study collected *A. calcoaceticus* isolates from the soil of two South African towns, which displayed antibiotic resistance to various classes of antibiotics, including cephalosporins, carbapenems, and tetracyclines (Stenstrom et al. 2016). However, further research into other soil-dwelling ACB complex species is needed to fully understand the spread of antibiotic-resistant pathogens in urban soil.

### Agricultural soil

Antibiotic-resistant ACB complex strains can be found in agricultural soil, reflecting the widespread use of antibiotics in modern agricultural practices. In Korea, where streptomycin is commonly used as a pesticide, *A. pittii* and *A. baumannii* resistant to streptomycin have been found in agricultural fields (Son et al. 2024). The isolates also displayed resistance to colistin, which is a common veterinary antibiotic (Kempf et al. 2016). Manure from animals treated with antibiotics represents a likely pathway through which antibiotic-resistant pathogens can enter the soil environment. To illustrate this, an experimental study found sulfonamide-resistant *A. baumannii* in soil two years after it was treated with pig slurry amended with sulfachloropyridazine (Byrne-Bailey et al. 2008). Additionally, resistance to clinically significant antibiotics have also been found in agricultural soil isolates. For instance, an *A. seifertii* isolate recovered from soil collected from a Brazilian corn field was found MDR to aminoglycosides, cephalosporins, and tetracycline (Furlan et al. 2019). Also, *A. baumannii* was found in agricultural soil samples in three different regions of Brazil where some isolates were MDR to extended‐spectrum cephalosporins, carbapenems, and tetracyclines (Furlan et al. 2018). It is unclear what the exact source of antibiotic resistance is in the Brazilian studies, however, agricultural soil resistance patterns appear to track with known local antibiotic use practices, such as in streptomycin resistance in Korean study. As compared to urban soil, agricultural soil resistance appears more mechanistically linked to identifiable antibiotic inputs, as seen in the persistence of sulfonamide resistance years after slurry application, demonstrating a direct, traceable rather than the more diffuse, and hard to specify resistance sources observed in urban soil. Overall, it appears that the prevalence and distribution of antibiotic-resistant ACB complex species in the soil environment are strongly influenced by human activities, underscoring the need for improved environmental surveillance and mitigation strategies.

## Animals

While the ACB complex is known as a pathogen to humans, they have also been found on healthy and infected animals, including fish, mammals, and birds (Muller et al. 2010, Rafei et al. 2015, Wilharm et al. 2017, Mitchell et al. 2018, Wang et al. 2020, Attili et al. 2024) (Table 1.4). Whether animals are a natural carrier of the ACB complex species or they are transiently colonized after interacting with other environmental reservoirs is unclear, as few studies have been conducted on healthy wild populations (Wilharm et al. 2017, Pereira et al. 2020).

### Companion animals

A few studies held on Reunion Island have identified healthy companion animals as carriers of *A. baumannii* (Belmonte et al. 2014, Pailhoriès et al. 2015). Although the isolates from companion animals were susceptible to most antibiotics, the majority belonged to clonal lineage that were commonly detected in human infections indicating genetic relatedness between isolates from the two host populations. Aside from *A. baumannii, A. pittii* and *A. calcoaceticus* have also been found on healthy canine skin (Mitchell et al. 2018). It is unclear if direct zoonotic transmission of the ACB complex species between humans and animals is frequent or clinically significant, but there have been a few documented cases in which genetically indistinguishable or highly related isolates were recovered from humans to animals, however, the frequency and source of transmission are unclear (Müller et al. 2014, Nocera et al. 2020). In one case study, the same *A. baumannii* strain isolated from an infected dog was also found asymptomatically colonizing the owner’s nasal cavity (Nocera et al. 2020). These study findings suggest that companion animals may serve less as an independent reservoir of resistant ACB complex species and more as an intermediary host reflecting ongoing exchange with the human-associated strain pool, a distinction that is difficult to resolve due to limited number of case reports and the absence of longitudinal surveillance data.

### Birds

A study in Poland found a diverse population of antibiotic-susceptible *A. baumannii* colonizing white stork nestlings (Wilharm et al. 2017). Strikingly, *A. baumannii* was recovered at a relatively high (25%) frequency while *A. pittii* was detected sporadically. However, the rate of isolation varied depending on the geographical location, and the researchers never recovered the same strain after sampling the same nest, suggesting *A. baumannii* more likely originated from the stork’s food and habitat rather than representing a stable colonizer (Wilharm et al. 2017). Additionally, other studies in Europe have found a low incidence of *A. baumannii* and *A. pittii* in wild birds with limited resistance to antibiotics (Łopińska et al. 2020, Grilli et al. 2025, Rapi et al. 2025). A few instances of MDR and carbapenem-resistant *A. baumannii* and *A. seifertii* genetically related strains reported in hospital settings were reported in captive and wild migratory aquatic birds at a Brazilian zoo (Narciso et al. 2017, 2020). These sporadic isolates may be attributed to the aquatic habitat of the birds, as Brazilian surface water has been shown to harbour carbapenem-resistant *A. baumannii* (Turano et al. 2016, Narciso et al. 2020). Similarly, *A. baumannii, A. pittii*, and *A. nosocomialis* were found in the wild pigeons’ droppings in cities, the dropping samples were also shown to carry carbapenemase-encoding genes, which may also reflect exposure to environments influenced by human activity, although the specific source was not determined (Morakchi et al. 2017). Although these studies show low antibiotic resistance, consistent with birds not being an intrinsic reservoir of the ACB complex, migratory birds and other wildlife could possibly play a role in the dissemination of antibiotic-resistant strains from contact with contaminated water or human waste (Greig et al. 2015, Dekić et al. 2018, Łopińska et al. 2020, Narciso et al. 2020).

### Fish

While most disease-causing *Acinetobacter* in fish are non-ACB complex species, such as *A. johnsonii* (Bi et al. 2023), *A. baumannii, A. pittii*, and *A. calcoaceticus* have recently been identified as emerging pathogens in fish, including loach, guppy and carp (Dekić et al. 2018, Wang et al. 2020). *A. pittii*, in particular, has been found highly virulent and pathogenic to fish, potentially threatening commercial cyprinid aquaculture operations (Wang et al. 2020). The report of a carp farm outbreak in India identified *A. pittii* as one of the causative agents, which was resistant to a variety of antibiotics, including ceftazidime and cefotaxime (Malick et al. 2020). Furthermore, MDR *A. baumannii* was detected in fish collected from a municipal dam in South Africa, along with other *Acinetobacter* spp. such as *A. pittii* and *A. calcoaceticus*, which were not tested for resistance (Anane et al. 2019). Similarly, in aquatic environments impacted by wastewater, *A. pittii* was found colonizing fish gills (Popović et al. 2022). A study found that colonization by *A. baumannii* occurs in freshwater fish if the concentration of *A. baumannii* is above a high threshold (Dekić et al. 2018). The colonization of *A. baumannii* in freshwater fish, together with the association between wastewater impacted water exhibit similar pattern observed in birds. This again indicates that a human-impacted environment could be the source of ACB complex species in animals (Dekić et al. 2018).

## Farm animals

ACB complex species have been found in farm animals, including cows, pigs, horses, donkeys, goats, chickens, and geese, as well as their associated environments (Rafei et al. 2015). A study found *A. baumannii* as a predominant airborne bacterium in a German duck hatchery (Martin and Jäckel 2011). In large scale animal production, antibiotics are commonly used for veterinary care, disease prevention and as growth promoting agents (Brown et al. 2017). These include antibiotics from classes which are also used to treat human infections such as tetracyclines, aminoglycosides, cephalosporins, and fluoroquinolones, which could create selective pressure for resistance conferring genes and the development of antibiotic resistance (Argudín et al. 2017, Chowdhury et al. 2021). In China, use of tetracyclines is widespread in the livestock industry, in this context, 8.9% of *Acinetobacter* isolates recovered from fecal and environmental samples on Chinese poultry farms were reported resistant to tigecycline and tetracycline (Cui et al. 2020). Similarly in Germany, *A. baumannii* isolated from 79.7% of meconium (fecal) samples from one day old turkey chicks were resistant to tetracycline, trimethoprim-sulfamethoxazole, ciprofloxacin and other veterinary fluoroquinolones (Schmitz et al. 2023). In contrast, a study from Scotland found that *A. baumannii* isolated from pig and cow fecal samples were susceptible to antibiotics such as tetracycline and ciprofloxacin, demonstrating variation in antimicrobial resistance among livestock-associated *A. baumannii* isolates, potentially reflecting differences in antibiotic use and agricultural practices across regions (Hamouda et al. 2011). Interestingly, rare occurrences of carbapenem-resistant *A. baumannii* have also been recorded in pig and cow feces (Webb et al. 2016, Hrenovic et al. 2019, Pulami et al. 2023). Although carbapenems are not permitted for use in livestock farming in any country, use of other beta-lactam antibiotics may create selection pressure favouring beta-lactamase genes that also happen to confer resistance to carbapenems (Webb et al. 2016). Another possibility is that these isolates could be the result of contamination from environmental or human sources that are yet undefined, highlighting a gap in the current research (Wang and Sun 2015, Hrenovic et al. 2019).

In contrast to environmental and fecal samples, *Acinetobacter* isolates from the body of healthy poultry and livestock have not been shown to demonstrate significant resistance to clinically relevant antibiotics (Wilharm et al. 2017, Chung et al. 2025). In Korea, *A. baumannii* isolates were recovered from pigs and cows, but only one animal isolate was resistant to a clinically significant antibiotic, tobramycin (Chung et al. 2025). Similarly, in a German study, only two *A. baumannii* isolates from the choana swabs of chicken and geese showed resistance to aminoglycoside antibiotics, and no isolates were found resistant to any other antibiotic classes that were also tested (Wilharm et al. 2017). Furthermore, another study conducted in Germany found all *A. baumannii* isolated from cow nasal, rectal, and fecal samples were susceptible to antibiotics currently used to treat *Acinetobacter* infection (Klotz et al. 2019). However, *A. baumannii* isolates in the German study were resistant to other 3^rd^ generation cephalosporins such as ceftiofur, a veterinary antibiotic commonly used in dairy farming. Use of 3^rd^ generation cephalosporins on these farms was associated with incidence of *A. baumannii* in cattle, indicating the selective pressure from antibiotic use could still contribute to the expansion of this clinically relevant species in livestock (Klotz et al. 2019).

Across various studies, resistance patterns appear to correspond with the use of specific antibiotic classes. For example, tetracycline resistance was prevalent in settings with heavy tetracycline use in China, while ceftiofur use in German dairy cattle was associated with increased resistance to cephalosporins. On the other hand, farms with lower or more regulated antibiotic use show largely susceptible isolates in Korea and Scotland. This clearly shows associations to the source of antibiotic usage in livestock farming industry. The presence of antibiotic-resistant ACB complex species in livestock manure should be cause for concern since manure is often applied to agricultural fields as fertilizer, allowing these pathogens to be introduced into the soil environment, where they contribute to the persistence of ARGs in the soil and can contaminate food crops (Leclercq et al. 2016, Ghaffar et al. 2025).

## Food

The ACB complex species have been found in all types of food and food products, including fish, meat, milk, cheese, and plant produce (Houang et al. 2001, Rafei et al. 2015). While *Acinetobacter* is not generally considered as a foodborne pathogen, food is one of the major points through which bacteria living in the environment can come into contact with humans (Malta et al. 2020).

### Meat production and slaughterhouses

In a German study, *A. baumannii, A. pittii*, and *A. calcoaceticus* were isolated from chicken and pig meat production settings (Savin et al. 2024). Resistance to cephalosporins was high in ACB complex isolates, while fluoroquinolone, trimethoprim-sulfamethoxazole, colistin, and carbapenem resistance was also occasionally observed (Savin et al. 2024). Interestingly, an *A. baumannii* strain found in the process water from poultry eviscerators carried a colistin resistance conferring plasmid which was genetically related to plasmids found in *A. baumannii* isolates from raw retail meat and clinical cases in other countries (Bitar et al. 2019, Savin et al. 2024). Similarly, a study from Korea found *A. baumannii* in the carcasses of cattle and pigs that were processed in slaughterhouses, which were resistant to ampicillin-sulbactam in addition to either tetracycline or tobramycin (Chung et al. 2025). Some *A. baumannii* isolates were also genetically related to *A. baumannii* previously isolated from cattle and pig farms (Chung et al. 2025). A study on slaughterhouse associated *Acinetobacter* strains from South Africa found *A. baumannii, A. pittii* and *A. calcoaceticus* in the slaughterhouse equipment and surroundings (Anane et al. 2019). In contrast to the Korean and German studies, the majority of *A. baumannii* isolates recovered were MDR (Anane et al. 2019). Additionally, the South African study found a less diverse *A. baumannii* population predominantly consisting of isolates assigned to ST1 and ST2 sequence types that have also been reported among local hospital isolates, whereas the other studies found a genetically diverse population and distinct from clinical strains (Anane et al. 2019, Chung et al. 2025). This suggests a potential epidemiological connection between food- and hospital-associated populations in South Africa, emphasizing the need for further surveillance of food production practices and transmission pathways. Differences in antibiotic use practices and hygiene standards in animal husbandry may contribute to the variation in findings between South Africa and other regions (Anane et al. 2019) (Table 1.5).

## Retail meat

The presence of the ACB complex species in raw meat has been well documented. In fact, single meat samples are often shown to carry multiple ACB complex species and multiple different clones of the same species (Carvalheira et al. 2017a, Hamze et al. 2025). In general, a diverse population of *Acinetobacter* spp. can be found in meat, reflecting the diverse population colonizing food-producing animals (Chung et al. 2025, Hamze et al. 2025). While some studies reported significant differences in the colonization of meat from different animals, with poultry and mutton being more frequently contaminated with *A. baumannii* compared to other types of meat (Lupo et al. 2014, Elbehiry et al. 2021), where other studies did not find any correlation (Carvalheira et al. 2017a). Findings have also differed regarding the level of antibiotic resistance in these strains. A study from Korea of local and imported meats using *Campylobacter* selective conditions instead found that *A. nosocomialis, A. seifertii, A. pittii*, and *A. baumannii* were highly prevalent (Cha et al. 2021). Two *A. nosocomialis* isolates from domestic pork were MDR, while all other isolates only displayed resistance to one or two antibiotics, including trimethoprim-sulfamethoxazole, colistin, gentamicin, or tetracycline (Cha et al. 2021). A study from Portugal identified *A. baumannii, A. pittii, A. seifertii, A. calcoaceticus* and *A. nosocomialis* in a variety of sampled meats, where 38.7% of the ACB complex isolates were MDR, and one *A. pittii* isolate was XDR (Carvalheira et al. 2017a). Interestingly, the prevalence of antibiotic resistance in non-ACB complex species was even higher than that of ACB complex species, suggesting the need for more research on these species as a reservoir of ARGs (Carvalheira et al. 2017a). In contrast, *A. baumannii, A. pittii*, and *A. dijkshoorniae* isolates from meat and fish by researchers in Peru, Lebanon, and India were all susceptible to the clinically relevant antibiotics tested (Rafei et al. 2015, Marí-Almirall et al. 2019, Vanishree et al. 2025). The differences in findings between these studies could be arising from the varying screening methodologies used to isolate strains and variation in the specific antibiotics tested. Additionally, studies conducted in different regions may yield different results based on local regulations and common practices regarding antibiotic use and meat processing. In support of this idea, similar antibiotic resistance patterns can be seen in meat from geographically close regions. For instance, in *A. baumannii* isolated from camel, chicken, sheep, and cow meat from Saudi Arabia, resistance to tetracycline, gentamicin, and tobramycin was more common compared to that to cephalosporins and carbapenems (Elbehiry et al. 2021). These results mirror a series of studies conducted in Iran, where high resistance to gentamicin, tetracycline, ciprofloxacin, and trimethoprim-sulfamethoxazole was observed in *A. baumannii* strains from meat samples, while carbapenem resistance was low (Tavakol et al. 2018, Askari et al. 2019a, 2019b). Likewise, a survey of seafood, including fish, lobster, and shrimp in Iran, also found a high level of resistance to gentamicin, tetracycline, and trimethoprim-sulfamethoxazole (Hasiri et al. 2023). On the other hand, in France and Switzerland, the majority of *A. baumannii* strains found in meat were susceptible to antibiotics with sporadic resistance to ciprofloxacin, colistin, and tetracycline (Lupo et al. 2014, Hamze et al. 2025). These findings demonstrate substantial regional variability in antimicrobial resistance profiles, with Saudi Arabia and Iran showing similar patterns of high gentamicin and tetracycline resistance and low carbapenem resistance, in contrast to the predominantly susceptible isolates reported in France and Switzerland. Such regional differences may be influenced by variation in local antibiotic use and regulatory practices.

## Dairy products

Similar to meat, food products such as milk and cheese can pose a potential risk to health through ACB complex species contamination, particularly since they are typically not cooked prior to consumption (Gurung et al. 2013) (Table 1.6). *Acinetobacter* has been reported as a dominant genus in raw milk, with the detection of *A. baumannii* and *A. calcoaceticus* in milk samples collected from Israeli farms. Although antibiotic resistance was not tested, the isolates were shown to display strong lipolytic activity and could hasten the spoilage of milk (Hantsis-Zacharov and Halpern 2007). Similarly, in Egypt, *A. baumannii* and *A. calcoaceticus* were also identified in raw milk, cheese, and cream, whereas *Acinetobacter* was not reported in pasteurized milk, highlighting the importance of the pasteurisation step in ensuring food safety (Saad et al. 2018). Another group in Egypt tested the antibiotic susceptibility of *A. baumannii* isolates in raw milk samples collected from local retail markets and found resistance to cefotaxime, ampicillin-sulbactam, and levofloxacin. However, some resistance to carbapenems was also observed, supported by the presence of carbapenem resistance-conferring genes such as *bla*_OXA-23_, *bla*_IMP_, and *bla*_NDM_ (Mohamed et al. 2022). Raw milk from Korea was also sampled, while the majority of *A. baumannii* isolates recovered were susceptible to antibiotics, some showed resistance to tetracyclines, trimethoprim-sulfamethoxazole, ampicillin-sulbactam, cephalosporins and aminoglycosides (Gurung et al. 2013). These Egyptian and Korean studies show variable resistance profiles without an obvious unifying driver, suggesting that resistance in raw dairy isolates may depend more on farm-level hygiene and individual retail supply chains than on any single environmental or regional factor.

### Fruits and vegetables

Fresh produce is another way through which the ACB complex species can enter human and clinical settings (Berlau et al. 1999) (Table 1.7). In Korea, a research group sampled crops from agricultural environments and recovered *A. seifertii* and *A. pittii* which were resistant to colistin, and positive for either *emrA* or *emrB* gene encoding efflux pumps (Son et al. 2024). These strains likely originated from the soil environment, as colistin-resistant *Acinetobacter* was also found in the soil where the crops were grown, suggesting a possible association between crop and soil populations. Furthermore, produce grown in or on soil has been shown to be significantly more likely to be contaminated by Gram-negative bacteria than those grown above ground (Zekar et al. 2017). A French group, tested unwashed fruits and vegetables, reported that antibiotic resistance was more prevalent in bacterial strains isolated from produce that was grown in contact with soil, suggesting soil may be the main source of antibiotic resistant bacteria on produce (Ruimy et al. 2010). This French study also found that *A. calcoaceticus* was the most prevalent *Acinetobacter* species on produce, though some *A. baumannii* strains were also isolated. Many isolates were resistant to cefotaxime and ceftazidime, while resistance to ticarcillin-clavulanic acid, piperacillin-tazobactam, and, very rarely, imipenem, was only observed in *A. calcoaceticus* (Ruimy et al. 2010). Studies using antibiotic-selective media from Algeria and Spain isolated *A. baumannii, A. pittii, A. calcoaceticus* and unsurprisingly reported high levels of resistance to cephalosporins and carbapenems, reflecting the selection method of the studies rather than true resistance rates (Zekar et al. 2017, Jiménez-Belenguer et al. 2023). Moreover, ready-to-eat salads sampled in Portugal using cefotaxime-containing media yielded low frequency of known ACB complex species, with the most prevalent species being *A. pittii*, followed by *A. calcoaceticus, A. geminorum*, and *A. lactucae*. Susceptibility to other antibiotics was not tested, but the isolates were screened for extended-spectrum beta-lactamase genes, and many were positive for *bla_ACC_, bla_SHV_, bla_DHA_* and *bla_VEB_* genes (Costa-Ribeiro et al. 2024). Another group in Portugal detected *A. calcoaceticus, A. pittii, A. baumannii, A. seifertii*, and *A. nosocomialis* on unwashed fruit and lettuce, where many of the isolates were MDR, with a number of them being XDR (Carvalheira et al. 2017). In fresh produce collected from retail markets in Jordan, researchers were able to identify *A. baumannii, A. pittii* and *A. nosocomialis*. Although the majority of the isolates were sensitive to most of the tested antibiotics, four isolates recovered from fruits and radish were classified as XDR. Two of the XDR *A. baumannii* strains were classified as novel or lesser-known sequence types, while the rest belonged to ST2, a globally prevalent clone commonly found in clinical sources (Ababneh et al. 2022b). The presence of these clones suggests that MDR and XDR strains found on produce can be genetically related to lineages commonly reported in clinical settings. However, further research is warranted to identify the route of transmission since genetic similarity alone does not demonstrate that these strains are transmitted between food and clinical environments. MDR ACB complex strains found in food have the potential to pose a public health risk, as they may cause community-acquired infections and facilitate transmission back into hospital settings (Berlau et al. 1999, Carvalheira et al. 2021).

## Conclusions and recommendations

Antibiotic-resistant ACB complex species can be found in all environmental niches, including water, soil, animals, and food (Turano et al. 2016, Tavakol et al. 2018, El Hafa et al. 2019, Hrenovic et al. 2019). While environmental ACB complex species such as *A. calcoaceticus* are found ubiquitously in water and soil, pathogenic MDR and carbapenem-resistant strains can be found near areas of heavy human activity, likely deriving from hospital and human waste (Hrenovic et al. 2017, Adewoyin et al. 2024). On the other hand, strains associated with animals are more susceptible to antibiotics compared to clinical isolates (Wilharm et al. 2017). Livestock animals often carry ACB complex strains that are resistant to veterinary antibiotics, but resistance to clinically relevant drugs is less common (Klotz et al. 2019). On meat, the effects of antibiotic use in animal husbandry can be more clearly seen, with resistance to antibiotics commonly used in animals, such as aminoglycosides, trimethoprim-sulfamethoxazole, and tetracyclines commonly observed (Islam et al. 2024). The natural environment boasts a genetically diverse range of ACB complex species, which can act as a reservoir of novel pathogenic clones and ARGs with the potential to threaten public health (Hernández-González and Castillo-Ramírez 2020). Additionally, antibiotic-susceptible ACB complex strains have the capacity to rapidly acquire resistance in clinical settings, further emphasizing the importance of understanding the prevalence and distribution of these species in the environment (Ababneh et al. 2022a, Hamze et al. 2025).

A key limitation across these studies is the reliance on culture-based methods and the extensive use of selective culture media containing antibiotic supplements for the isolation of ACB complex strains. Culture-based methods only recover a subset of culturable ACB complex strains rather than full range of ACB complex isolates. Additionally, since these media are specifically designed to enrich for antibiotic resistant strains, resistance frequencies reported from various studies which used selective isolation methods cannot be interpreted as estimates of baseline resistance prevalence and compared to other study findings where non-selective isolation methods were used. Future studies should be designed by considering quantifying resistance prevalence relative to populations recovered through non-selective isolation methods. Resistance estimates derived from selective media methods should be interpreted as evidence of the presence and distribution of resistance determinants within an enriched subpopulation, rather than as epidemiologically representative measures of resistance prevalence.

The dissemination of antibiotic-resistant ACB complex strains in the environment poses a significant “One Health” concern (Hernández-González and Castillo-Ramírez 2020). Human activity and waste contribute to the prevalence of pathogenic ACB complex strains in soil environments (Hrenovic et al. 2017, Tavakol et al. 2018, El Hafa et al. 2019, Hrenovic et al. 2019). Animals such as white storks, which interact with these environments, can become transiently colonized by the ACB complex species (Wilharm et al. 2017). Similarly, antibiotic-resistant strains from hospitals, slaughterhouses, and municipal WTPs can persist through wastewater treatment and enter local waters, affecting wildlife such as fish, and potentially spread through the environment on migratory birds, however, genetic and epidemiological evidence indicate the connectivity and the direction of movement are unclear and need further research (Hrenovic et al. 2016, Narciso et al. 2020, Popović et al. 2022). Moreover, the same water can be used by farmers as irrigation for their crops, leading to contamination of soil and food (Carvalheira et al. 2017). In some developing countries, untreated wastewater is used for irrigation, which poses an even greater risk for public health (Heyde et al. 2025). Use of animal manure as fertilizer can also introduce ARGs and resistant ACB complex strains into crop fields (Breyer et al. 2026). These strains residing in soil can infiltrate human and hospital environments through fresh produce, where they have the potential to cause pneumonia, bacteremia, soft tissue and skin infections (Berlau et al. 1999, Visca et al. 2011). Additionally, other agricultural products such as meat, fish, and animal products can also harbour antibiotic-resistant ACB complex strains and pose a threat to public health (Ababneh et al. 2022a, Mohamed et al. 2022, Hasiri et al. 2023). Finally, antibiotic residue and resistant ACB complex strains from crop fields and livestock farming can pollute nearby waters through runoff and leach into groundwater, completing the cycle (Gao et al. 2023, Benoit et al. 2024, Khmaissa et al. 2024).

Overall, the research findings demonstrate that the widespread presence of antibiotic-resistant ACB complex species and strains circulating across every major environmental compartment, including water, soil, animals, and food, creating multiple pathways for human exposure. Their movement among clinical, agricultural, and natural settings reflects a highly interconnected “One Health” system in which human activity, wastewater management practices, and antibiotic use influence the distribution and evolution of resistance. The environment not only serves as a reservoir for diverse ACB complex species and novel resistance genes but also acts as a conduit through which ACB complex species and strains can re–enter hospitals or acquire additional resistance traits. Understanding how these organisms persist and spread across ecological boundaries is, therefore, essential for designing effective monitoring strategies, implementing antibiotic stewardship, and improving wastewater treatment across clinical, agricultural, and environmental sectors in order to mitigate the emergence of new pathogenic lineages. Finally, as it has already been suggested for the non-human populations of *A. baumannii* (Castillo-Ramírez et al. 2025), close attention needs to be paid to the under studied virulence potential of environmental ACB complex species/strains and their potential to cause human infections. Continued research, particularly on lesser-known ACB complex species, is critical for anticipating future public health risks and interrupting transmission pathways that contribute to environmental and foodborne contamination.
